# Implementing interpersonal psychotherapy globally: a content analysis from 31 countries

**DOI:** 10.1017/S0033291724003003

**Published:** 2024-12

**Authors:** Jennifer J. Mootz, Myrna M. Weissman

**Affiliations:** 1 Vagelos College of Physicians and Surgeons, Columbia University Department of Psychiatry, New York, USA; 2 Translational Epidemiology and Mental Health Equity, New York State Psychiatric Institute, New York City, USA

**Keywords:** Africa, Asia, consolidated framework for implementation research, Europe, implementation, interpersonal counseling, interpersonal psychotherapy, North America, Oceania, United Kingdom

## Abstract

While there is ample evidence for the efficacy of IPT, confirmed through the results of the efficacy review, on the ground implementation factors are less well understood. We compiled a book on the global reach of IPT by requesting contributions from local authors through word-of-mouth methods. This approach resulted in reports from 31 countries across six continents and 15 diverse populations within the US that spanned the age range and types of usage. In this paper, our aim was to collate and summarize book contributors' descriptions of barriers and facilitators as related to their experiences of implementing IPT across the 31 countries. We conducted a conceptual content analysis and then applied the updated Consolidated Framework of Implementation Research (CFIR) to deductively organize the barriers and facilitators into its five domains. Most found IPT to be relevant and acceptable and described minor variations needed for tailoring to context. National level policies and mental health stigma were highlighted in the outer setting. Availability of specialists and general and mental health infrastructure were considerations relevant to the inner setting. Many sites had successfully implemented IPT through delivery by nonspecialized providers, although provider workload and burnout were common. Clients faced numerous practical challenges in accessing weekly care. Primary strategies to mitigate these challenges were use of telehealth delivery and shortening of the intervention duration. Most programs ensured competency through a combination of didactic training and case supervision. The latter was identified as time-intensive and costly.

## Background

A review of efficacy studies of Interpersonal Psychotherapy (IPT) for depression appears in this issue of Psychological Medicine (Cohen et al., 2024). Like all other tested psychotherapies and like medication as well, IPT is not efficacious for all patients with major depression or even the same patient at different episodes. However, there are sufficient data from controlled clinical trials to confirm that IPT is one of the efficacious evidence-based psychotherapies. In this paper, we briefly describe its implementation in a wide range of countries and conditions as fully reported in our recent book (Weissman & Mootz, 2024), available as an open access resource, with descriptions from 31 countries.

## IPT in brief

IPT has been described in several manuals for depression and other disorders (Weissman, Markowitz, & Klerman, 2018). Simplified versions, using the same content, called interpersonal counseling IPC, have also appeared, proposed at first for use by providers without specialized training in mental health (Weissman et al., 2014). IPT is based on the observation that whatever the ‘cause’ of depression, biological, environmental, and likely some combination, the onset of symptoms occurs in an interpersonal situation. These situations are called problem areas and include grief, interpersonal disagreements, life changes, or loneliness. Identifying the emergence of symptoms in association with the current problem[s] and finding ways to deal with the problems is the basis of IPT.

The use of IPT in low-income countries began after a clinical trial was carried out in Uganda, the results of which were published in 2000 (Bolton et al., 2003). Sean Mayberry, a foreign service officer in Uganda, read this paper and realized that many Ugandan citizens fared less well with health and development initiatives given the debilitating effects of depression on help-seeking and treatment adherence. He estimated that 20–30% of people in malaria and HIV/AIDS programs did not respond to treatment because they may have been depressed. Mayberry went on to found StrongMinds, a nonprofit organization dedicated to providing community-based, accessible mental health care with group IPT delivered by community health workers and peer volunteers. StrongMinds has treated 600 000 people in Uganda and Zambia and has recently initiated programming in the US.

As IPT originated in a high-income country, examining how its principles resonate in diverse settings can reveal vital insights into cultural variations in interpersonal relationships, social support systems, and community structures. This comprehensive understanding can lead to more culturally sensitive adaptations of the therapy, ensuring that it meets the needs of varied populations effectively. Currently, over 150 clinical trials of IPT have been carried out. While there is ample evidence for the efficacy of IPT, confirmed through the results of the efficacy review, on the ground implementation factors are less well understood. Exploring implementation factors – such as training, resource availability, and community acceptance – will help identify barriers and facilitators, ultimately enhancing the accessibility of IPT worldwide.

## Methods

**Figure 1.**
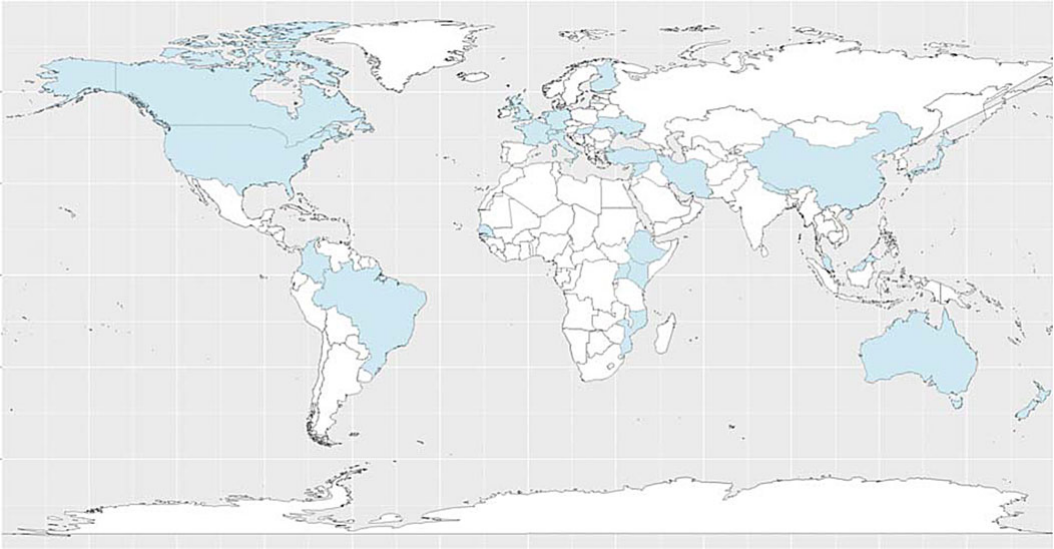
World map of represented countries. *Note*. Alphabetized list of represented countries: Australia (Reay, 2024), Brazil (Mello et al., 2024), Canada (Grigoriadis et al., 2024; Ravitz et al., 2024), China (Zheng et al., 2024a, 2024b), Colombia (Espinel et al., 2024), Ethiopia (Wondimagegn et al., 2024), Finland (Law & Ranta, 2024b), France (Omay, Glatigny-Dallay, Lavigne, Salomé, & Sengelen, 2024), Germany (Brakemeier, 2024), Hong Kong (Chung, 2024), Hungary (Stauder & Novák, 2024), Iran (Rafiei Alhosaini & Rezaei-Jamalouei, 2024), Israel (Klomek et al., 2024), Italy (Bellino & Bozzatello, 2024), Japan (Mizushima, 2024), Kenya (Meffert, 2024; Yator & Kumar, 2024), Lebanon (Verdeli et al., 2024), Malaysia (Pereira & Verghis, 2024), Mozambique (Khan et al., 2024), Nepal (Pradhan, Rose-Clarke, Verdely, & Shrestha, 2024), Netherlands (Dozeman et al., 2024; Peeters, Jonker, & Blom, 2024), New Zealand (Luty & Nolan, 2024), Scotland (Graham & Irvine Fitzpatrick, 2024), Senegal (Ziadeh et al., 2024), Switzerland (Hovaguimian & Omay, 2024), Turkey (Aydın, Omay, Öztürk, & Nur Ekşi, 2024), Uganda (Mayberry, 2024), UK (Law, 2024; Law & Duffy, 2024a), Ukraine (Law, Klymchuk, & Gorbunova, 2024), US (Chiao et al., 2024; Dietz, 2024; Heckman et al., 2024; Johnson, 2024; Kidd et al., 2024; Markowitz, 2024; Mufson et al., 2024; Patel et al., 2024; Reynolds, 2024; Swartz et al., 2024; Wesemann & Judge-Ellis, 2024).

We recently edited a book on the global reach of IPT (available as an open access resource) to learn more about how IPT has been adapted and implemented globally. We had many questions, often not covered in the research literature, about whether the concepts were relevant and understandable in different parts of the world; how IPT is being implemented and adapted; obstacles to and what works well in implementation; and how training, supervision, and monitoring are done. We sent requests for book chapter contributions by email (see Supplementary Document 1 for a sample email) to colleagues known for their work in IPT and others found through a literature search about use of IPT globally, word-of-mouth, and through posting on the International Society of IPT listserv. This unsystematic journey resulted in reports from 31 countries (Fig. 1) across six continents and 15 diverse populations within the US that spanned the age range and types of usage (Weissman & Mootz, 2024).

In this paper, our aim was to collate and summarize book contributors' descriptions of barriers and facilitators as related to their experiences of implementing IPT across the 31 countries. We applied the revised and updated Consolidated Framework of Implementation Research (CFIR) to deductively organize the barriers and facilitators into its five domains: innovation (what is being implemented), outer setting (factors outside the site of implementation), inner setting (where the innovation is being implemented), individuals (IPT deliverers and recipients), and implementation process (Damschroder, Reardon, Widerquist, & Lowery, 2022).

## Analysis

We conducted a conceptual content analysis, an analysis method that focuses on identifying existence and patterns of communication in text (Drisco & Maschi, 2016). We reread chapters several times and extracted any information that pertained to the CFIR domains of implementation (Damschroder et al., 2022) into a Word table organized by the chapters (rows) and CFIR domains (columns). Several chapters have specific sections dedicated to describing barriers and facilitators, given our request for authors to cover that content. In our reading, we focused on extracting information from those sections but also read the chapters in their entirety to identify other content that may be relevant. We placed all applicable information about barriers and facilitators into Table 1. We then reviewed Table 1 by CFIR domain to determine themes of barriers and facilitators experienced across contexts for each domain. The overarching themes are described in the results.

**Table 1.** Barrers and facilitators of implementing interpersonal psychotherapy and its variations globally according to the consolidated framework for implementation research (CFIR)

CFIR Domain	Facilitators	Barriers
Innovation	Culturally syntonic and relevant; positive symptom outcomes; delivered for free and in local languages; use of songs, visual aids, conversation starters, ice breakers, incentives; adapted for phone-based delivery during COVID; deployed WhatsApp chatbot to identify symptoms and enroll; used measurement to determine intervention duration; offered in groups to address stigma; incorporated local sayings, idioms, phrases, and concepts; easy to adapt; easy to train nonspecialized workforce; session dose lessened for feasibility; group modality helped reduce isolation; formation of same-sex groups improved HIV stigma; alternate forms of delivery (e.g. online) needed; shifted from diagnosis- to symptom-based screening; involved family, parents, and partners; incorporated social media, photos, and meaningful items into therapy; accounted for relational hierarchies and local social structures; encouraged locally congruent mourning for grief cases; session dose increased for bullying and comorbid disorders; family buy-in important for adolescents; applied human rights framework; addressed psychosocial needs first; expanded biopsychosocial model; included religion/spirituality; included historical, political, and environmental factors; easily implemented in telehealth; adaptable for different populations; local models of wellbeing used for adaptation; developmental modifications made; included work stress; incorporated Cultural Formulation interview; time limit can be motivating; used maintenance sessions; technology or telehealth reduces barriers	No initial adaptation for a local setting or population; length of time of session (too long); lack of visual cues if telephone delivered
Outer Setting	Engaging policy and community stakeholders; linking participants to community-based support upon termination; creating anti-stigma videos and ads shown in health centers; psychotherapy recognized as evidence-based; psychotherapy/IPT reimbursable; demand for psychotherapy increased with economic development; ISIPT chapter established; local language materials with local case examples; formal certification supported provider motivation; forming a peer network for providers; give education lectures in implementation setting; IPT adopted as recommended evidence-base treatment for depression; adequate insurance coverage; IPC in homes enhanced participation and retention; recommend to collaborate with academics to test outcomes; international collaborations	Mental health and other stigma (e.g. HIV) common; novelty of talk therapy in clinical culture; counseling not covered by health insurance; lack of child protection services; lack of legal protections for refugees and asylum seekers; policies limited work and educational opportunities for clients; sparsely populated rural areas hard to cover; colonization and historical trauma created mistrust in systems; laws that limit professional responsibilities or ability to provide services; government partners reluctant to recruit; IPT not included in national guidelines; poor cell phone or internet coverage
Inner setting	Over-including trainees helpful to mitigate dropout and increase community education; offered flexible starting times; developed feasible safety protocols; having a champion who encouraged peers; having well established mental health care services already in place; having access to specialized care and consultation; demonstrated good attendance in school settings; delivered care in primary care, community health settings, and obstetrics and gynecology offices	Limited health workers and specialists; resistance from health administrators; patient identification, service integration, treatment accessibility; need for bilingual therapists and supervisors; providers also exposed to adversity and hardship; referral processes for severe cases not in place or effective; implementation geography limited; integrating IPT delivery with other service sectors; lack of basic infrastructure for meeting; lack of childcare resources; other interventions (e.g. CBT, medication) had more in-setting saturation
Individuals: Providers	Use of task-sharing (lay and peer facilitators, nurses, community health workers, primary care providers) common; peers receptive to training and have connections to communities; having two facilitators gave task-shifted providers confidence; having providers of varying ages and co-led by man and woman helpful; provider willingness to implement; having interactive and dynamic trainers; having trainers who speak local languages; observing improvement in clients' symptoms motivating; opportunity for professional growth; interpersonal competencies helped facilitation; social work background helped; community health workers felt empowered; improved ability to identify and facilitate linkage to care; recommended that providers be aware of cultural needs, practice cultural humility, be aware of bias and privilege, use decolonizing research strategies, respect and use cultural wisdom practices, explore and address barriers to treatment;	Hierarchical relationship between provider and client where advice given; professional workload burden; trainee experience varied widely; diagnostic burden on specialist; worker dissatisfaction, burnout; provider concern about lack of evidence in local population; professional development path unclear; reticence to participate in international English language training; reluctant to focus on skill development through use of roleplay, decision and communication analysis; supervisors not experienced in an intervention version; used materials in a second language; people involved with implementation had other jobs and little time; linguistic, cultural, and and/or ethno-racial matching clinician/client unavailable.
Individuals: Recipients	IPT concepts accepted and relevant; telephone delivery highly acceptable	Had to travel long distances; weekly sessions not feasible; clients wanted to resolve problems quickly; cost a concern when not covered by government or insurance; stigma internalized by patients and family members; attrition rates high; barriers such as transport, childcare, lack of time, family interference, lack of employer permission faced; high exposure to traumatic stressors; some clients saw the time-limited treatment as a form of abandonment; gender roles can inhibit participation (e.g. women expected to prioritize family needs before own)
Process: Training/Supervision	Didactic training followed by supervision; increased supervision period; incorporated training on vicarious trauma; provided ongoing refresher trainings; trained foundations with mhGAP; gave training for supervisors; offered or recommended giving training in postgraduate or other training programs; created teaching videos; developed supporting materials (e.g. session checklists); used train the trainer/apprenticeship model; applied active learning; incorporated local case examples; used technology to support providers; emphasized adaptation in training and supervision; support given to facilitators when they became distressed with content such as suicidality and abuse; core competencies taught and measured; used a competency-based framework and matched training to correspond	Distance supervision from other countries not successful; translated trainings and supervision meetings required time and translating complex concepts sometimes difficult; in person supervision not scalable and feasible; training demand couldn't be met with supervision; cost of supervision

## Results

### Innovation

Many authors described IPT as culturally congruent and noted appreciation for the relational approach and framework to mental distress and wellbeing. This trend was especially evident among authors working in more relationally oriented cultures (e.g. Kenya, Ethiopia, China, Latinx in the US (Patel, Mufson, & Lewis-Fernández, 2024; Wondimagegn, Hailu, Ravitz, & Pain, 2024; Yator & Kumar, 2024; Zheng et al., 2024a, 2024b)). Overall, contributors found IPT relatively easy to learn, simple to adapt, relevant for everyday problems, and a straightforward intervention to train to non-specialists.

IPT specialists have adapted the intervention to work with many diverse populations globally, for example, people living with HIV (Ziadeh et al., 2024), girls and women who experience gender-based violence (Meffert, 2024) (especially in sub-Saharan Africa but also with forcibly displaced populations in Malaysia and Colombia (Espinel, Shultz, & Verdeli, 2024; Pereira & Verghis, 2024)), perinatal adolescent girls and women (Swartz, Curran, & Grote, 2024), and all stages of life from preschool to older age (Dietz, 2024; Klomek, Latzer, & Hera, 2024; Mufson, Klomek, Garcia, & Makridis, 2024; Reynolds, 2024).

Many interventionists delivered IPT to treat common mental disorders (depression, anxiety, PTSD) through a more comprehensive transdiagnostic lens rather than focus solely on depression. This broader application was especially likely to occur in low-resource settings, such as Mozambique and other countries in sub-Saharan Africa (Khan et al., 2024). Other mental health and health problems addressed were somatoform disorder, adjustment disorder, bipolar disorder, eating disorders, perinatal mental health, comorbid medical illnesses, ADHD, autism spectrum, schizophrenia, sexual violence, nutritional counseling, and suicide prevention.

Most contributors found that the four core interpersonal problem areas (grief, life changes, disagreements, and loneliness) were applicable for their contexts and made minor adjustments to ensure IPT and its variants were congruent with local values and customs. For example, regarding grief, authors ensured that mourning rituals and behaviors were appropriate to context. In some cultures and religious orientations (e.g. Muslim clients in Senegal), for instance, it is not considered acceptable to cry for the deceased (Ziadeh et al., 2024). For disputes, authors discussed the importance of respecting and working within longstanding social structures and adopting the constellations and conceptions of family (e.g. polygamy) that were relevant. Others, especially those working with indigenous populations in Australia, New Zealand, and the US, extended the biopsychosocial model to include historical trauma, culture, religion and spirituality, and connection to land (Brave Heart, Chase, Elkins, & Martin, 2024; Luty & Nolan, 2024; Reay, 2024). Life changes could encompass developmental transitions traumatic events, such as being forcibly displaced. Disputes could also occur with organizations or host communities for the forcibly displaced (e.g. in Colombia (Espinel et al., 2024)). Loneliness and social isolation could occur in the context of discrimination.

Authors highlighted several facilitators for implementation. These facilitators included ensuring accessibility by offering the intervention for no cost (e.g. Uganda (Mayberry, 2024)). Another facilitator was tailoring the intervention to be culturally congruent through delivery in local languages, incorporation of local sayings and conceptions of mental health, use of locally relevant visual aids and songs, and inclusion of developmentally appropriate activities. Including family members, such as intimate partners, parents, in-laws, or elders, was additionally facilitative. Including family members was especially mentioned when working with younger populations, in the context of working with the dispute problem area, and important in settings with sophisticated social structures and hierarchies. Most authors shortened the duration of treatment, citing feasibility for health systems and clients as the primary rationale. A few programs increased the number of sessions to address other mental illnesses (e.g. borderline personality disorder in Italy (Bellino & Bozzatello, 2024)) or adverse experiences (e.g. being bullied in Japan (Mizushima, 2024)). The adolescent model, interpersonal psychotherapy for adolescents (IPT-A), was extended to work with young adults in some settings (Mizushima, 2024).

### Outer setting

Several factors pertinent to the outer setting were present. These implementation topics included health insurance coverage, policies pertaining to mental health practice, stigma of mental health problems, and geographic challenges.

One of the principal barriers reported across settings was national policies for insurance coverage for delivery of and participation in IPT. In some settings, for example, mainland China, mental health counseling was not covered by health insurance (Zheng et al., 2024a, 2024b). In others, IPT as a specific intervention was not included in the guidelines for what was allowable treatment approaches for addressing mental disorders. For example, in Japan, the National Health Insurance Plan did not cover IPT and specialists who were not trained as medical doctors were not permitted to provide diagnostic and intervention services without supervision from a medical doctor (Mizushima, 2024). In other countries, such as Brazil, mental health had not been included in the national health strategic plan (Mello, Matsuzaka, & Sweetland, 2024). Some regulatory bodies have limited professional activities that make task-sharing for mental health responsibilities less feasible.

Those who lived in countries that had policies that increased access to care, such as Lebanon and Australia, viewed those policies as important facilitators (Reay, 2024; Verdeli, Clougherty, Sardana, Sönmez, & Maradian, 2024). There was a recognition that when ministries of public health explicitly included mental health in their strategic programming, this facilitated mental health care broadly. Some pointed to new laws that required that interventions be evidence-based, an opportunity to advocate for inclusion of IPT given its strong research foundation. Other facilitative policies allowed for the costs of psychotherapy to be covered as a part of medical care and individual and group sessions covered by public insurance. In some settings, IPT has been recommended as an approved treatment for depression. Also germane to regulation, several authors mentioned that establishment of national ISIPT chapters helped synthesize efforts for dissemination. Potential for formal certification through ISIPT has served as a motivator for providers. Several noted that implementation started with translation of manuals that included local case examples and books and these activities were important facilitators.

Another central barrier discussed across many countries and settings was stigma related to mental health conditions. There were various levels of familiarity with psychotherapy as an intervention. The novelty of this approach, as opposed to a more medical approach with psychopharmacology, was sometimes met with suspicion (e.g. Ethiopia) (Wondimagegn et al., 2024). Mental health stigma amplified for people living with co-occurring stigmatized medical conditions, such as HIV, and for minoritized communities, such as those who identify as sexual or gender minorities (Heckman, Anderson, & Heckman, 2024; Kidd, Kaczmarkiewicz, Langer, Koljack, & Hughes, 2024). Others pointed to the detrimental effects of colonization and historical trauma and oppressive histories that have perpetrated atrocities and left marginalized communities mistrustful of health service systems. Authors, such as those from Malaysia working with refugees, described insufficient legal protections for forcibly displaced populations that exacerbated conditions and led to a further marginalized status (Pereira & Verghis, 2024). Forcibly displaced people could experience exploitation and discrimination from host communities. Some policies were noted to hinder people's access to economic opportunities, heightening challenges and intensifying distress during significant life changes.

To address stigma, several people incorporated community awareness and education into their programming or made recommendations to do so. For instance, in Ethiopia, the authors constructed videos to show in primary care waiting rooms to provide education about depression and recovery (Wondimagegn et al., 2024). In Uganda, StrongMinds developed a chatbot that could be accessed with a quick response (QR) code that would give information about depression and help link people to care, if needed (Mayberry, 2024). Others mentioned that delivering IPT in group modality promoted connections among group members and helped people feel less isolated and stigmatized. Some elected to use local idioms of distress to help de-stigmatize mental health problems. Several programs have implemented IPT into the school system to enhance community trust and improve access. As part of the implementation package, education lectures have been delivered for teachers, students, and heads of schools to build awareness and knowledge and support for students' participation.

Geographic constitution impeded implementation in some settings. This was especially noted by contributors working in rural areas that were sparsely populated. They described having difficulty providing adequate coverage for those living in remote settings. People and providers in rural settings were said to experience challenges with cell coverage and transportation issues coupled with long distances between sites were prohibitive for attending weekly sessions.

### Inner setting

Some implementation barriers and facilitators related to the inner organizational setting were identified. Having limited local human resources, particularly mental health specialists, was cited as a burden. High workloads may be placed on staff and as a result, burnout and dissatisfaction with the professional role and responsibilities was more likely. Authors in Senegal mentioned that there was a diagnostic burden on the psychiatrist who evaluated potential participants for diagnostic eligibility to enter the IPT group study (Ziadeh et al., 2024). They surmised that shifting from diagnostic- to symptom-based measurement would support feasibility going forward. Contributors working in Colombia expressed similar concern that referral processes and linkage to care for severe cases was sometimes challenging; only 25% of referred people in their study received specialized care (Espinel et al., 2024). Contributors sometimes cited resistance from administrators that hindered their work. Integrating IPT with other service sectors was occasionally described as challenging.

Approaches that worked well for programs were implementing IPT with flexible start times, accommodating schedules of participants and IPT trainees, and not rushing through session agendas. The latter was especially salient in places where emotional expression may not be customary (e.g. China) (Zheng et al., 2024a, 2024b). Another strategy found to be helpful was careful development of clear and feasible safety protocols. Relatedly, having access to a psychiatrist for specialized care, either on site or on a consultancy basis was helpful. Including more trainees in programs could be a potential solution to address lack of fit in some instances and potential burnout. Additionally, having well established mental health care services provided the infrastructure needed for successful implementation of IPT. However, those from low-resource settings regularly pointed out challenges corresponding to basic infrastructure, such as a need for private meeting spaces or provision of childcare services for clients.

## Individuals

### Providers

Many sites implemented task-sharing, the training of non-specialists to deliver mental health interventions and most described this implementation strategy as successful. In Uganda and Zambia, IPT groups were led by lay and peer facilitators who demonstrated receptivity to training and were optimal intervention deliverers given their strong connections to communities (Mayberry, 2024). Other settings, too, noted willingness among task-shared providers to learn and implement IPT. In Brazil, for instance, the authors reported that community health workers expressed feeling empowered and they showed an improved ability to identify mental health problems and facilitate linkage to care (Mello et al., 2024). In Israel, there was high teacher satisfaction for the social emotional learning program that incorporated IPT concepts and was taught to children as young as preschool (Klomek et al., 2024). In Colombia, peer internally displaced women were selected to deliver IPT to enhance sustainability, and they demonstrated excellent adherence to the intervention model (Espinel et al., 2024). There were several places, such as Colombia, where providers were also exposed to traumatic stressors, adversity, and hardship and needed to relocate for economic or safety reasons (Brave Heart et al., 2024; Espinel et al., 2024). Providing additional support and including content on vicarious trauma in training were methods to address provider needs. When working with indigenous populations in the US, authors recommended that tailored support should be offered to providers (Brave Heart et al., 2024). Giving support to providers who could become distressed at content, such as suicidality and abuse, was also an important strategy. While most thought that task-sharing was successful, one site mentioned that having some background in social work was thought to have helped (Ziadeh et al., 2024). In a minority of settings, there was initial provider skepticism about the efficacy of IPT and concern about the lack of evidence of IPT for working with local populations but, for instance in Hong Kong, acceptability improved after a series of workshops took place (Chung, 2024). Trainees were occasionally noted to be potentially reticent to participate in international, English language trainings if they had less fluency in English. People involved with implementation had other responsibilities, often fulltime jobs, and their time was limited.

Some mentioned provider qualities including those who demonstrated strong interpersonal skills and ability to work in teams, as facilitators. For example, in Mozambique, authors identified that having interactive and dynamic trainers helped facilitate learning (Khan et al., 2024). It also was pointed out in other settings that therapists be aware of cultural needs and that adaptation to cultural context should be emphasized in training and supervision. Advice for working with Indigenous populations in the US was for providers to practice with cultural humility, be aware of their biases and privilege, use theoretical and cultural wisdom practices, and respect and recognize the intelligence and wisdom of Indigenous people (Brave Heart et al., 2024). In Finland, supervisors observed a reluctance to focus on skill development through some of the commonly used IPT techniques of roleplay, decision and communication analysis (Law & Ranta, 2024b). They reported that targeted training and supervision successfully increased providers' sense of self-efficacy and adoption. In cultures and professional roles where advice-giving is a common and expected practice, some noted that training helped providers be less directive and prescriptive. Others noted that having two leaders for groups supported non-specialized providers' confidence. They also thought that having group leaders of varying ages was helpful to address diverse group members' needs. Having organizational and administrative support and provision of opportunities for certification and professional growth was facilitative in that it improved provider morale and motivation.

### Intervention recipients

Several settings observed high rates of common mental disorders and exposure to significant adversity. In Colombia, for instance, internally displaced participants reported an average of 24 exposures to pre-displacement trauma, peri-displacement loss, and post-displacement life changes and severe symptom levels (Espinel et al., 2024). Ensuring that care is trauma informed and recognizing the traumatic stressors that many communities have undergone can improve the relevance and contextual tailoring of services.

Contributors identified many barriers to clients' accessing care. Some examples of these challenges were transportation issues and travel costs, difficulty obtaining time away from work, family interference, lack of time, and childcare needs. As a result of these difficulties, some authors reported high attrition rates. For these reasons, many programs reduced the number of sessions to support feasibility in face of these challenges given weekly attendance over the course of many weeks or months was difficult to impossible. In China, clients preferred to resolve problems quickly due to competing demands (Zheng et al., 2024a, 2024b). Cost was a concern in settings where IPT was not covered by insurance. On top of these practical challenges, stigma internalized by patients and family members was pervasive and mentioned by many contributors, as described as a challenge occurring in the outer setting. Several programs described implementing IPT through telephone or telehealth and these modalities were thought to improve attrition rates and reduce some of the barriers to attending sessions (Grigoriadis, Dennis, & Ravitz, 2024; Heckman et al., 2024).

Regarding construction of IPT groups, authors in Senegal found that forming same-sex groups helped reduce stigma and enable discussion about HIV-related problems (Ziadeh et al., 2024). In some countries, many dialects and languages could be present which may also affect facilitation of groups and group members' ability to communicate clearly with one another. Linking participants to community-based support upon termination was another identified facilitator.

## Process

### Training and supervision

Most sites described teaching as initiating with a didactic training with attendance requirements that ranged from two days to two weeks, which was followed by extensive weekly or monthly supervision where trainees would present and receive feedback and support in adherence to the IPT model. Some countries, such as Finland, The Netherlands, and Canada had developed online or virtual options for didactic delivery or self-study (Dozeman, Donker, Schotanus, & Van Schaik, 2024; Law & Ranta, 2024b; Ravitz et al., 2024). For larger scale-up initiatives, implementers frequently used a train the trainer model. For example, in Ethiopia, psychiatrists trained psychiatric nurses and psychologists who then trained primary care nurses (Wondimagegn et al., 2024). Occasionally examinations of didactic content were given to assess knowledge acquisition, but most thought it was the provision of continuous supportive supervision that was the primary facilitator for implementation. For trainees who were not mental health specialists, introductory training on mental health problems, such as the mhGAP, was also included as well as training in group facilitation, core skills, and safety and protection procedures. Additional training for supervisors was sometimes offered. One observed challenge in training was when there was wide variability in trainee background and experience. For competency, some stipulated a number of cases (typically 2–3) that needed to be completed with adherence to the model. The number of cases generally increased (e.g. to 5) for those who wanted to supervise. Annual trainings, advanced trainings, role-play workshops, and refresher courses also included to improve implementation. Some programs established ongoing peer supervision meetings.

Many countries described needing to bring in external, internationally based consultants (usually English speakers) when initiating nationwide dissemination of IPT. Several of these authors talked about barriers of having English-speaking supervisors. The need for translation was a burden on time, for instance, and created potential for miscommunication regarding cultural elements of case presentation. Having local change experts who helped with adaptation, forming a peer network, and developing local training materials and workshops and case reports facilitated dissemination. Several countries, such as Mozambique and Lebanon, noted building a local cadre of supervisors and trainers was imperative (Khan et al., 2024; Verdeli et al., 2024).

Several people recommended that IPT be implemented in graduate and medical (e.g. psychiatry, nursing) training programs. Costs and lack of available certified supervisors affected scalability of dissemination beyond individual programs and research studies (e.g. Brazil) (Mello et al., 2024). It was challenging for several sites to provide supervision to meet the training demand. Some, for instance, authors in UK, noted that when variants of IPT (e.g. interpersonal counseling for adolescents) were scaled-up that it was difficult to identify supervisors who had experience with that modification (Law & Duffy, 2024a). Others noted that using a competency-based framework and matching training to correspond to the framework helped enhance quality.

## Discussion

To our knowledge, this is the first paper to describe barriers and facilitators of implementing IPT from a global perspective. Book contributions represented 31 countries with many diverse populations, including diverse developmental stages, forcibly displaced, perinatal women, veterans, people living with HIV, and minoritized populations, among others. Authors described several prominent barriers and facilitators across four CFIR domains.

Despite the unsystematic sampling method, there were some common threads that we have described. There was surprising widespread acceptance of the core components and problem areas associated with distress: grief from death, disagreements, life changes, and a paucity of relationships. These problem areas and their acceptance across regions, cultures, diverse populations, and socioeconomic levels are consistent with the global writings on the importance of human connection. For example, the World Health Organization recently established a Commission on Social Connection to address loneliness as a public health priority through national policy to individual level psychological treatments (WHO, 2024). The US Surgeon General has declared loneliness and social isolation a public health emergency and developed several initiatives to combat this growing problem (Murthy, 2023).

In the outer setting, factors such as policies regarding provision of mental health services, insurance coverage (often connected to policies), and stigma were important factors raised by many authors irrespective of location. Jenkins et al., noted a central challenge to global mental health efforts and funding has been a lack of internationally agreed upon indicators for mental health (Jenkins, Baingana, Ahmad, McDaid, & Atun, 2011). Community-based and social mechanisms that support mental health solutions may seem more complex than treatment programs for health-related problems, such as HIV/AIDS, TB, and malaria prevention. A separate analysis of government spending on mental health in 78 countries concluded that around 25% of governments allocated less than 1% to mental health and found an association between burden of infectious disease and mental health spending (Rajkumar, 2022). Thus, countries where the infectious disease burden is high may view health spending to offer more benefit, although the bidirectional link between health and mental health has been well established (Doherty et al., 2013; Fabrazzo et al., 2023; Yang et al., 2020).

Some contributors identified challenges of not having enough locally based human resources to implement IPT. The beginning stages of national scale-up processes often required having externally based consultants provide initial training and supervision to build the first local cadre of IPT professionals. Challenges of language and cultural differences presented during training and supervision. For this reason, successfully building a national cadre of local professionals was paramount. Others have similarly commented on the challenges of scaling up psychological treatments due to barriers in being able to scale training and supervision activities (Fairburn & Patel, 2014). Digital training has been proposed as a potential solution to support scale-up (Fairburn & Patel, 2014). We would add that digital trainings should be offered and developed in multiple languages. Having nonspecialist providers deliver mental health interventions through task-sharing was commonly employed to address the obstacle of not having enough specialist capacity to meet the population need. While this strategy has been largely employed in LMICs, there has been movement to implement this strategy in high-income countries as well (Lange, 2021). We anticipate that this will continue to expand and further examination of determining severity stratification to optimize stepped care approaches (i.e. who improves with brief interventions delivered by task-shared providers and who requires specialty care) will be helpful. Several contributors described task-sharing as successful and noted strong ability to be adherent to the intervention model.

At the individual level, some settings noted an initial skepticism regarding IPT and its fit and effectiveness with local providers. However, training and cultural tailoring reduced this skepticism. Being able to deploy interventions with cultural humility and sensitivity and adapting the intervention culturally were cited as important provider attributes. Respectful attitudes and communication of providers has shown to be associated with recovery outcomes of empowerment, connectedness, hope, life satisfaction, and internalized stigma in a US population (Wong et al., 2019). To train and evaluate providers in core therapeutic competencies, Kohrt et al., developed the ENhancing Assessment of Common Therapeutic factors (ENACT) rating scale in the context of a task-sharing program in Nepal (Kohrt et al., 2015). From a review of the literature, they identified several attributes to include in the scale. Some examples of items are nonjudgmental communication, displays of warmth and empathy, reflective listening, and rapport building (Kohrt et al., 2015). Continuing to operationalize wellness promoting provider attributes and communication across global settings and identifying best strategies for training providers in these skills would be fruitful.

Several contributors highlighted a range of barriers to accessing care. In rural areas, transportation was frequently noted as a challenge. Some had successfully employed telephone or digital means of delivery which has helped address this problem. Almost in totality, contributors shortened the length of treatment duration given clients' inability to attend numerous weekly sessions. Most still found interventions to be effective. Future studies should evaluate minimum dosage of interventions needed, maintenance of gains following briefer treatments, and develop a better understanding of how to tailor dosage to client presentation and context.

## Limitations

There are many limitations of this analysis. Thirty-one countries constitute a fraction of the world. Some regions are underrepresented (e.g. South America) and others not represented at all. The methods used for country selection were through word-of-mouth, advertising on the ISIPT listserv, and a review of literature and thus may not be representative or generalizable to other populations. Most contributions had not systematically studied implementation factors. Rather, their reports were based on anecdotal observations from often extensive experience initiating or scaling up IPT nationally. Other limitations are our categories. We chose CFIR as it is widely used in implementation research, including in low- and middle-income countries. We could have reviewed barriers and facilitators by region or socioeconomic status. We leave others to do that, if interested.

## Conclusion

IPT is just one of several evidence-based therapies. Like all interventions, IPT is not efficacious in all conditions for all populations. Since the first randomized controlled trial of IPT in Uganda, implementation of IPT has rapidly expanded globally. This review of implementation and adaptation of IPT in 31 countries has presented some commonly experienced barriers and facilitators according to the CFIR domains. Common among the contributions was the general acceptance of the core interpersonal problem areas and the idea that disruptions in human attachments associated with distress.

## Supporting information

Mootz and Weissman supplementary material Mootz and Weissman supplementary material

## Supplementary material

The supplementary material for this article can be found at https://doi.org/10.1017/S0033291724003003.

## References

[ref1] Aydın, N., Omay, O., Öztürk, N., & Nur Ekşi, B. (2024). Interpersonal psychotherapy training in Turkey. In M. M. Weissman, & J. J. Mootz (Eds.), Interpersonal psychotherapy: A global reach (pp. 100–105). New York: Oxford University Press.

[ref2] Bellino, S., & Bozzatello, P. (2024). Interpersonal psychotherapy in Italy. In M. M. Weissman, & Mootz J. J. (Eds.), Interpersonal psychotherapy: A global reach (pp. 257–264). New York City: Oxford University Press.

[ref3] Bolton, P., Bass, J., Neugebauer, R., Verdeli, H., Clougherty, K., Wickramaratne, P, … Weissman, M. (2003). Group interpersonal therapy for depression in rural Uganda: A randomized controlled trial. JAMA, 289(23), 3117–3124. 12813117 10.1001/jama.289.23.3117

[ref4] Brakemeier, E.-L. (2024). Interpersonal psychotherapy in Germany. In M. M. Weissman, & J. J. Mootz (Eds.), Interpersonal psychotherapy: A global reach (pp. 239–250). New York City: Oxford University Press.

[ref5] Brave Heart, M. Y. H., Chase, J., Elkins, J., & Martin, J. (2024). A culturally grounded interpersonal psychotherapy with American Indians/Alaska natives. In M. M. Weissman, & J. J. Mootz (Eds.). Interpersonal psychotherapy: A global reach (pp. 398–404). New York City: Oxford University Press.

[ref6] Chiao, S., Tepper, M., Sweetland, A. C., Mootz, J. J., Clougherty, K. F., Steele, J. L., … Wainberg, M.L. (2024). ENGAGE NYC: Interpersonal counseling in New York city community settings. In M. M. Weissman, & J. J. Mootz (Eds.), Interpersonal psychotherapy: A global reach (pp. 475–486). New York City: Oxford University Press.

[ref7] Chung, J. P.-Y. (2024). Interpersonal psychotherapy in Hong Kong. In M. M. Weissman, & J. M. Mootz (Eds.), Interpersonal psychotherapy: A global reach (pp. 196–201). New York City: Oxford University Press.

[ref8] Cohen, Z., Breunese, J., Markowitz, J., Weitz, E., Hollon, S., Browne, D., … Driessen, E. (2024). Comparative efficacy of interpersonal psychotherapy and antidepressant medication for adult depression: A systematic review and individual participant data meta-analysis. Psychological Medicine, 54(14), 3785–3794. 10.1017/S0033291724001788 PMC11578913 39494789

[ref9] Damschroder, L. J., Reardon, C. M., Widerquist, M. A. O., & Lowery, J. (2022). The updated consolidated framework for implementation research based on user feedback. Implementation Science, 171 [Internet]. [cited 2023 Mar 28]; 17(1), 1–16. Available from: https://implementationscience.biomedcentral.com/articles/10.1186/s13012-022-01245-0 36309746 10.1186/s13012-022-01245-0 PMC9617234

[ref10] Dietz, L. (2024). Family-Based interpersonal psychotherapy (FB-IBT) for depressed preadolescents. In M. M. Weissman, & J. J. Mootz (Eds.), Interpersonal psychotherapy: A global reach (pp. 361–370). New York City: Oxford University Press.

[ref11] Doherty, A. M., Kelly, J., McDonald, C., O'Dywer, A. M., Keane, J., & Cooney, J. (2013). A review of the interplay between tuberculosis and mental health. General Hospital Psychiatry, 35(4), 398–406. 23660587 10.1016/j.genhosppsych.2013.03.018

[ref12] Dozeman, E., Donker, T., Schotanus, A. Y., & Van Schaik, A. (2024). Internet-Delivered interpersonal psychotherapy (i-IPT) in the Netherlands. In M. M. Weissman, & J. J. Mootz (Eds.), Interpersonal psychotherapy: A global reach (pp. 271–277). New York City: Oxford University Press.

[ref13] Drisco, J., & Maschi, T. (2016). Content analysis. New York City: Oxford University Press.

[ref14] Espinel, Z., Shultz, J. M., & Verdeli, H. (2024) Interpersonal counseling for internally displaced women in Bogotá, Colombia. In M. M. Weissman, & J. J. Mootz (Eds.), Interpersonal psychotherapy: A global reach (pp. 351–360). New York City: Oxford University Press.

[ref15] Fabrazzo, M., Cipolla, S., Pisaturo, M., Camerlengo, A., Bucci, P., Pezzella, P., … Galderisi, S. (2023). Bidirectional relationship between HIV/HBV infection and comorbid depression and/or anxiety: A systematic review on shared biological mechanisms. Journal of Personalized Medicine, [Internet]. [cited 2024 Jun 24]; 13(12), 1689. Available from: /pmc/articles/PMC10744606/38138916 10.3390/jpm13121689 PMC10744606

[ref16] Fairburn, C. G., & Patel, V. (2014). The global dissemination of psychological treatments: A road map for research and practice. American Journal of Psychiatry, [Internet]. [cited 2024 Jun 24]; 171(5):495–498. Available from: https://ajp.psychiatryonline.org/doi/10.1176/appi.ajp.2013.13111546 24788281 10.1176/appi.ajp.2013.13111546

[ref17] Graham, P., & Irvine Fitzpatrick, L. (2024). Interpersonal psychotherapy in different populations in Scotland. In M. M. Weissman, & J. J. Mootz (Eds.), Interpersonal psychotherapy: A global reach (pp. 278–284). New York City: Oxford University Press.

[ref18] Grigoriadis, S., Dennis, C.-L., & Ravitz, P. (2024) Telephone interpersonal psychotherapy delivered by nurses for postpartum depression. In M. M. Weissman, & J. J. Mootz (Eds.), Interpersonal psychotherapy: A global reach (pp. 422–430). New York City: Oxford University Press.

[ref19] Heckman, T. G., Anderson, T., & Heckman, B. D. (2024). Telephone interpersonal psychotherapy (tele-IPT) for rural persons with HIV and comorbid depression. In M. M. Weissman, & J. J. Mootz (Eds.), Interpersonal psychotherapy: A global reach (pp. 454–461). New York City: Oxford University Press.

[ref20] Hovaguimian, T., & Omay, O. (2024) Interpersonal psychotherapy training in Switzerland. In M. M. Weissman, & J. J. Mootz (Eds.), Interpersonal psychotherapy: A global reach (pp. 94–99). New York: Oxford University Press.

[ref21] Jenkins, R., Baingana, F., Ahmad, R., McDaid, D., & Atun, R. (2011). International and national policy challenges in mental health [Internet]. Vol. 8, Mental Health in Family Medicine. Radcliffe Publishing and Wonca; [cited 2024 Jun 22]. pp. 101–114. Available from: /pmc/articles/PMC3178192/PMC3178192 22654973

[ref22] Johnson, J. E. (2024). Interpersonal psychotherapy in prisons and jails for Major depression. In M. M. Weissman, & J. J. Mootz (Eds.), Interpersonal psychotherapy: A global reach (pp. 462–474). New York City: Oxford University Press.

[ref23] Khan, S., Feliciano, P., Wainberg, M. L., Suleman, A., Santos, P., Ngozo, D., … Mootz, J. J. (2024). Interpersonal counseling scale-up in Mozambique. In M. M. Wiessman, & J. J. Mootz (Eds.), Interpersonal psychotherapy: A global reach (pp. 158–168). New York City: Oxford University Press.

[ref24] Kidd, J. D., Kaczmarkiewicz, R., Langer, S. J., Koljack, C., & Hughes, T. L. (2024). Interpersonal psychotherapy with sexual and gender minority individuals. In M. M. Weissman, & J. J. Mootz (Eds.), Interpersonal psychotherapy: A global reach (pp. 444–453). New York City: Oxford University Press.

[ref25] Klomek, A. B., Latzer, Y., & Hera, R. (2024). Interpersonal psychotherapy in Israel. In M.M. Weissman, & Mootz J. J. (Eds.), Interpersonal psychotherapy: A global reach (pp. 309–316). New York City: Oxford University Press.

[ref26] Kohrt, B. A., Jordans, M. J. D., Rai, S., Shrestha, P., Luitel, N. P., Ramaiya, M. K., … Patel, V. (2015). Therapist competence in global mental health: Development of the ENhancing assessment of common therapeutic factors (ENACT) rating scale. Behaviour Research and Therapy, [Internet]. [cited 2021 Apr 7]; 69, 11–21. Available from: /pmc/articles/PMC4686771/25847276 10.1016/j.brat.2015.03.009 PMC4686771

[ref27] Lange, K. W. (2021). Task sharing in psychotherapy as a viable global mental health approach in resource-poor countries and also in high-resource settings. Glob Heal J., 5(3), 120–127.

[ref28] Law, R. (2024). Guided self-help interpersonal psychotherapy in the United Kingdom. In M. M. Weissman, & J. J. Mootz (Eds.), Interpersonal psychotherapy: A global reach (pp. 290–296). New York City: Oxford University Press.

[ref29] Law, R., & Duffy, F. (2024a). Interpersonal psychotherapy training and accreditation in the United Kingdom. In M. M. Weissman, & J. J. Mootz (Eds.), Interpersonal psychotherapy: A global reach (pp. 106–117). New York: Oxford University Press.

[ref30] Law, R., & Ranta, K. (2024b). Interpersonal psychotherapy in Finland. In M. M. Weissman, & J. J. Mootz (Eds.), Interpersonal psychotherapy: A global reach (pp. 230–238). New York City: Oxford University Press.

[ref31] Law, R., Klymchuk, V., & Gorbunova, V. (2024) Interpersonal psychotherapy in Ukraine. In M. M. Weissman, & J. J. Mootz (Eds.), Interpersonal psychotherapy: A global reach (pp. 285–289). New York City: Oxford University Press.

[ref32] Luty, S., & Nolan, D. (2024). Interpersonal psychotherapy in New Zealand. In M. M. Weissman, & J. J. Mootz (Eds.), Interpersonal psychotherapy: A global reach (pp. 335–342). New York City: Oxford University Press.

[ref33] Markowitz, J. C. (2024). Interpersonal psychotherapy for post-traumatic stress disorder (PTSD). In M. M. Weissman, & J. J. Mootz (Eds.), Interpersonal psychotherapy: A global reach. (pp. 439–443). New York City: Oxford University Press.

[ref34] Mayberry, S. (2024). Interpersonal psychotherapy group: A scalable solution to the depression epidemic in Zambia and Uganda. In M. M. Weissman, & J. J. Mootz (Eds.), Interpersonal psychotherapy: A global reach (pp. 178–186). New York City: Oxford University Press.

[ref35] Meffert, S. (2024). Interpersonal psychotherapy in east Africa: The Nyanza Region of Kenya. In M. M. Weissman, & J. J. Mootz (Eds.), Interpersonal psychotherapy: A global reach (pp. 151–157). New York: Oxford University Press.

[ref36] Mello, M. F., Matsuzaka, C., & Sweetland, A. C. (2024). Interpersonal counseling in Brazil: A task shift experience. In M. M. Weissman, & J. J. Mootz (Eds.), Interpersonal psychotherapy: A global reach (pp. 343–350). New York City: Oxford University Press.

[ref37] Mizushima, H. (2024). Interpersonal psychotherapy in Japan. In M. M. Weissman, & J. J. Mootz (Eds.), Interpersonal psychotherapy: A global reach (pp. 202–206). New York City: Oxford University Press.

[ref38] Mufson, L., Klomek, A. B., Garcia, G., & Makridis, V. (2024). Interpersonal psychotherapy for adolescents (IPT-A). In M. M. Weissman, & J. J. Mootz (Eds.), Interpersonal psychotherapy: A global reach (pp. 371–381). New York City: Oxford University Press.

[ref39] Murthy, V. H. (2023). Our epidemic of loneliness and isolation: The U.S. surgeon general's advisory on the healing effects of social connection and community. [cited 2024 Jun 24]; pp. 1–82. Available from: https://www.hhs.gov/sites/default/files/surgeon-general-social-connection-advisory.pdf

[ref40] Omay, O. J.-M. S., Glatigny-Dallay, E., Lavigne, B., Salomé, N., & Sengelen, J.-M. (2024). Interpersonal psychotherapy training in France. In M. M. Weissman & J. J. Mootz (Eds.), Interpersonal psychotherapy: A global reach (pp. 85–93). New York: Oxford University Press.

[ref41] Patel, S. R., Mufson, L., & Lewis-Fernández, R. (2024). Interpersonal psychotherapy with Hispanic/Latinx individuals. In M. M. Weissman, & J. J. Mootz (Eds.), Interpersonal psychotherapy: A global reach (pp. 405–413). New York City: Oxford University Press.

[ref42] Peeters, F., Jonker, K., & Blom, M. (2024). Interpersonal psychotherapy in the Netherlands. In M. M. Weissman, & J. J. Mootz (Eds.), Interpersonal psychotherapy: A global reach (pp. 266–270). New York City: Oxford University Press.

[ref43] Pereira, X. V., & Verghis, S. (2024) Interpersonal psychotherapy for refugees in Malaysia. In M. M. Weissman, & J. J. Mootz (Eds.), Interpersonal psychotherapy: A global reach (pp. 207–216). New York City: Oxford University Press.

[ref44] Pradhan, I., Rose-Clarke, K., Verdely, H., & Shrestha, P. (2024). Group interpersonal psychotherapy for adolescents in Nepal. In M. M. Weissman, & J. J. Mootz (Eds.), Interpersonal psychotherapy: A global reach. New York City: Oxford University Press. pp. 217–226.

[ref45] Rafiei Alhosaini, N., & Rezaei-Jamalouei, H. (2024). Interpersonal psychotherapy in Iran. In M. M. Weissman, & J. J. Mootz (Eds.), Interpersonal psychotherapy: A global reach (pp. 299–308). New York City: Oxford University Press.

[ref46] Rajkumar, R. P. (2022). The correlates of government expenditure on mental health services: An analysis of data from 78 countries and regions. Cureus, 14(8), e28284. doi:10.7759/cureus.28284 36039126 PMC9400922

[ref47] Ravitz, P., Sittampalam, S., Bäck, M., Croswell, K., Swartz, H. A., & Radha Singla, D. (2024). Interpersonal psychotherapy training – digital, online educational formats. In M. M. Weissman, & J. J. Mootz (Eds.), Interpersonal psychotherapy: A global reach (pp. 65–72). New York: Oxford University Press.

[ref48] Reay, R. E. (2024). Interpersonal psychotherapy in Australia. In M. J. Weissman, & J. J. Mootz (Eds.), Interpersonal psychotherapy: A global reach (pp. 327–334). New York City: Oxford University Press.

[ref49] Reynolds, C. F. I. (2024). Interpersonal psychotherapy in older adults with Major depression. In M. M. Weissman, & J. J. Mootz (Eds.), Interpersonal psychotherapy: A global reach (pp. 393–397). New York City: Oxford University Press.

[ref50] Stauder, A., & Novák, M. (2024). Interpersonal psychotherapy in Hungary. In M. M. Weissman, & J. J. Mootz (Eds.), Interpersonal psychotherapy: A global reach (pp. 251–256). New York City: Oxford University Press.

[ref51] Swartz, H. A., Curran, M., & Grote, N. K. (2024). Brief interpersonal psychotherapy (IPT-B) for perinatal depression. In M. M. Weissman, & J. J. Mootz (Eds.), Interpersonal psychotherapy: A global reach (pp. 414–421). New York City: Oxford University Press.

[ref52] Verdeli, H., Clougherty, K. F., Sardana, S., Sönmez, C. C., & Maradian, S. P. (2024). Interpersonal psychotherapy for Lebanese and Syrian refugees in Lebanon. In M. M. Weissman, & J. J. Mootz (Eds.), Interpersonal psychotherapy: A global reach (pp. 317–326). New York City: Oxford University Press.

[ref53] Weissman, M., & Mootz, J. (Eds.) (2024). Interpersonal psychotherapy: A global reach [internet]. NY: Oxford University Press, Open Access. Available from: https://fdslive.oup.com/www.oup.com/academic/pdf/openaccess/9780197652084.pdf

[ref54] Weissman, M., Markowitz, J., & Klerman, G. (2018). The guide to interpersonal psychotherapy: Updated and expanded version. New York, NY: Oxford University Press.

[ref55] Weissman, M. M., Hankerson, S. H., Scorza, P., Olfson, M., Verdeli, H., Shea, S., … Wainberg, M. (2014). Interpersonal counseling (IPC) for depression in primary care. American Journal of Psychotherapy, 68(4), 359–383. 26453343 10.1176/appi.psychotherapy.2014.68.4.359 PMC4603528

[ref56] Wesemann, D., & Judge-Ellis, T. (2024). Interpersonal psychotherapy delivered by nurses. In M. M. Weissman, & J. J. Mootz (Eds.), Interpersonal psychotherapy: A global reach (pp. 431–438). New York City: Oxford University Press.

[ref57] WHO. (2024). WHO COMMISSION ON SOCIAL CONNECTION. WHO [Internet]. [cited 2024 Jun 24]; Available from: https://www.who.int/groups/commission-on-social-connection

[ref58] Wondimagegn, D., Hailu, H., Ravitz, P., & Pain, C. (2024) Interpersonal psychotherapy in Ethiopia (IPT- E). In M. M. Weissman, & J. J. Mootz (Eds.), Interpersonal psychotherapy: A global reach (pp. 135–142). New York: Oxford University Press.

[ref59] Wong, E. C., Collins, R. L., Breslau, J., Burnam, M. A., Cefalu, M. S., & Roth, E. (2019). Associations between provider communication and personal recovery outcomes. BMC Psychiatry, [Internet]. [cited 2024 Jun 24]; 19(1), 1–8. Available from: https://bmcpsychiatry.biomedcentral.com/articles/10.1186/s12888-019-2084-9 30922292 10.1186/s12888-019-2084-9 PMC6439978

[ref60] Yang, H., Chen, W., Hu, Y., Chen, Y., Zeng, Y., Sun, Y., … Song, H. (2020). Pre-pandemic psychiatric disorders and risk of COVID-19: A UK biobank cohort analysis. Lancet Heal Longev, [Internet]. [cited 2024 Jun 24]; 1(2), e69–e79. Available from: http://www.thelancet.com/article/S2666756820300131/fulltext 10.1016/S2666-7568(20)30013-1 PMC7832159 33521769

[ref61] Yator, O., & Kumar, M. (2024). Interpersonal psychotherapy in Kenya. In M. M. Weissman, & J. J. Mootz (Eds.), Interpersonal psychotherapy: A global reach (pp. 143–150). New York: Oxford University Press.

[ref62] Zheng, W., Li, W., Luo, Y., Huang, M., Sun, X., & Zhou, X. (2024a). Interpersonal psychotherapy in mainland China: The rapidly growing practice. In M. M. Wiessman, & J. M. Mootz (Eds.), Interpersonal psychotherapy: A global reach (pp. 189–195). New York City: Oxford University Press.

[ref63] Zheng, W., Liu, X., Huang, M., & Li, W. (2024b). Interpersonal psychotherapy training in mainland China. In M. M. Weissman, & J. J. Mootz (Eds.), Interpersonal psychotherapy: A global reach (pp. 79–84). New York: Oxford University Press.

[ref64] Ziadeh, S., Bernard, C., Ndiaye, I., & Seydi, M. (2024). Interpersonal psychotherapy group in Senegal: First steps and future plans. In M. M. Weissman, & J. J. Mootz (Eds.), Interpersonal psychotherapy: A global reach (pp. 169–177). New York City: Oxford University Press.

