# Supplementary material for: Implementing interpersonal psychotherapy globally: a content analysis from 31 countries
Source: Psychol Med. 2024 Dec 16;54(16):4493–502. doi: 10.1017/S0033291724003003 (PMC11779550; doi:10.1017/S0033291724003003)
Supplement: Mootz and Weissman supplementary material [file S0033291724003003sup001.docx]

**Supplementary Document 1**

Dear Author,

You are an expert in many areas, and I would like to ask you about one of them, IPT. The material below explains the context of the request.

*Interpersonal Psychotherapy: A Global Reach*

Myrna M. Weissman, PhD, & Jennifer Mootz, PhD

 ​

­­­­­­­­­­­­­­­­­­­­­­Book Description

*Interpersonal Psychotherapy: A Global Reach*describes the rapidly expanding global dissemination of IPT, which has occurred over the last 5 years, and was accelerated during the pandemic. Many of these developments, are underreported in scientific journals. Our prime interest is in the practical use and adaptation of IPT around the world, including training adaptations for community health workers; the use of new technologies; as well as the expansion of IPT to address disorders in addition to depression. Global experts from multiple countries have been invited to describe the implementation of IPT in their settings. By now, there are close to 200 clinical trials of the various forms of IPT. While we are interested in these results, this book focuses on the adaptation of IPT in diverse settings, populations and the experiences of experts who have participated in these developments.

**Guidelines for Authors**

You are being asked to contribute to this book because of your expertise in adapting and/or implementing IPT in global settings. I hope you will contribute a chapter on your experience in using IPT in [fill in country or setting]. Your contributions can be between 1500 and 3000 words. You would be listed as an author and can invite others. We would like this chapter by **May 1^st^, 2022.**

1. Describe the context of your work, geographic area and population (age, gender, diagnosis, etc.).

2. Describe adaptations of IPT that were necessary

- Describe IPT and adaptations (duration, intensity, treatment targets)
- What adaptations to training or supervision were required, and you recommend?

3. Explain barriers and facilitators of implementation

- What were challenges in implementation of IPT? How were challenges addressed?
- What helped implementation work well?

4. What are your future plans? What do you recommend?

5. Provide a typical case example.

- Choose some aspect of IPT you would like to illustrate, such as a problem area, the sick role, interpersonal inventory, and present it in a case.

6. Are there any publications, either yours or others, describing this work? If the manual has been translated in your language, let us know.

**Could you let us know by February 15, 2022, if you are interested in participating or have interest but for different ideas?**

Sincerely,

Myrna Weissman

Jennifer Mootz
